# Gene body DNA methylation in seagrasses: inter- and intraspecific differences and interaction with transcriptome plasticity under heat stress

**DOI:** 10.1038/s41598-021-93606-w

**Published:** 2021-07-12

**Authors:** Laura Entrambasaguas, Miriam Ruocco, Koen J. F. Verhoeven, Gabriele Procaccini, Lazaro Marín-Guirao

**Affiliations:** 1 Integrative Marine Ecology Department, Stazione Zoologica Anton Dohrn, Villa Comunale, 80121 Napoli, Italy; 2 Terrestrial Ecology Department, Netherlands Institute of Ecology (NIOO-KNAW), Droevendaalsesteeg 10, 6708 PB Wageningen, The Netherlands; 3 Seagrass Ecology Group, Oceanographic Center of Murcia, Spanish Institute of Oceanography, C/Varadero, 30740 San Pedro del Pinatar, Spain

**Keywords:** Ecological genetics, Marine biology, Evolutionary genetics

## Abstract

The role of DNA methylation and its interaction with gene expression and transcriptome plasticity is poorly understood, and current insight comes mainly from studies in very few model plant species. Here, we study gene body DNA methylation (gbM) and gene expression patterns in ecotypes from contrasting thermal environments of two marine plants with contrasting life history strategies in order to explore the potential role epigenetic mechanisms could play in gene plasticity and responsiveness to heat stress. In silico transcriptome analysis of CpG_O/E_ ratios suggested that the bulk of *Posidonia oceanica* and *Cymodocea nodosa* genes possess high levels of intragenic methylation. We also observed a correlation between gbM and gene expression flexibility: genes with low DNA methylation tend to show flexible gene expression and plasticity under changing conditions. Furthermore, the empirical determination of global DNA methylation (5-mC) showed patterns of intra and inter-specific divergence that suggests a link between methylation level and the plants’ latitude of origin and life history. Although we cannot discern whether gbM regulates gene expression or vice versa, or if other molecular mechanisms play a role in facilitating transcriptome responsiveness, our findings point to the existence of a relationship between gene responsiveness and gbM patterns in marine plants.

## Introduction

Human-induced climate change is challenging the persistence of natural ecosystems. The rapid environmental changes that organisms are currently facing, are forcing them to acclimatize, adapt or migrate to avoid becoming extinct. Unlike slow genetic adaptation of populations through natural selection, phenotypic plasticity of individual genotypes facilitates a fast response to environmental change and it is currently considered key for the ecological success of organisms in a rapidly changing world^1–3^. An important component of the ability of an organism to change its phenotype is the modulation of gene expression (e.g.^4,5^). The mechanisms by which gene expression, and thus phenotypic plasticity, are influenced and regulated remain poorly understood in most organisms. Epigenetic changes (e.g. DNA methylation and histone modifications) have been suggested as key candidates for regulating gene expression and generating/regulating phenotypic plasticity^6–10^ and adaptive responses to environmental change (e.g.^11^).

Cytosine methylation within DNA is an evolutionary widespread epigenetic modification found in most eukaryotes, including plants and animals^12^. In plants, DNA methylation is found at cytosines (C) in three sequence contexts: CG, CHG, and CHH, where H corresponds to C, T, or A, and it is established and maintained by different pathways^13^. Methylated CG (mCG) is the most abundant form of mC in plant genomes and predominantly occurs in noncoding regions such as transposable elements and other repetitive DNA regions^14,15^. Protein coding regions are also methylated but its establishment, maintenance, possible function and evolutionary consequences remain unclear. Gene body DNA methylation (gbM) refers to genes with enriched mCG within the transcribed regions and depletion at the transcriptional start and termination sites^16,17^. GbM is a common feature of eukaryotic genomes and remains well conserved among plants and animals^13,18^. Furthermore, gbM genes are often long (bp), slowly evolving and evolutionary conserved when compared to genes with low gbM; in contrast to CHG or CHH methylation, CG methylation is the only correlated across orthologues although the cause of this correlation remains unknown^19–21^.

Although the causes and potential functional consequences of gbM for gene expression are not fully understood^22^, genic CG methylation was reported to be associated with gene expression in both plants and animals^17^. Several studies have found that methylation of gene bodies varies according to gene function, indicating that highly conserved genes with housekeeping functions tend to be more heavily methylated than those with inducible functions^15,21,23–26^. Thereby, contrary to the transcriptionally repressive effects of other chromatin modifications within gene bodies, gbM genes are typically moderately or constitutively expressed. One hypothesis is that the main function of gbM is most likely homeostatic e.g. by enhancing splicing accuracy, reducing the accumulation of histone variant H2A.Z and/or establishing constitutive expression patterns within housekeeping genes^15,17,26–29^. Yet, the absence of methylation within gene bodies could allow a variety of transcriptional opportunities in genes involved in stress and environmental responses^24,30^. In invertebrates, low gbM in environmental responsive genes seem to increase phenotypic plasticity and the adaptive potential of species^18,24,25,31,32^. Nevertheless, gbM may also lack functional consequences for gene expression^21^, and its complete loss in some basal plants^21^ and in the angiosperm *Eutrema salsugineum*^16^ demands for further studies on the general function of this type of DNA methylation in plants.

In the absence of direct methylation data, the normalized CpG content (i.e. the ratio of observed to expected CpG, CpG_O/E_) is a robust measure of the strength of DNA methylation levels on an evolutionary time scale^33^. The correlation between (historic) DNA methylation and normalized CpG content is based on the mutagenic properties of mC, which undergoes spontaneous deamination to thymine more readily than normal cytosine^33^. Because of this hypermutability, sequences that are heavily methylated in the germline become deficient in CpGs over evolutionary time^34^. Consequently, genomic regions that are subject to heavy germline DNA methylation (hypermethylated) lose CpG dinucleotides over time and have lower-than-expected CpG_O/E_. In contrast, regions that undergo little germline DNA methylation (hypomethylated) maintain high CpG_O/E_. In addition to capturing historic information on DNA methylation, CpG_O/E_ levels can be used as a proxy for *current* DNA methylation in the genomic region of interest^35–37^ because CpG_O/E_ strongly correlates with direct measures of somatic DNA methylation^38^. This has been shown in many animals^18,24,25,37,39,40^ and plants^20,21^.

Seagrasses are a polyphyletic group of angiosperms fully adapted to develop their life cycle completely submerged in marine waters, for which they have lost some biological characteristics of their terrestrial ancestors and acquired others from seaweeds^41^. This group of marine clonal plants forms extensive meadows in coastal bottoms of almost all continents, where they provide high-value ecological and economic services^42^, including coastal protection, nutrient cycling, water quality improvement, fishery maintenance and carbon sequestration, among others^43^. One of the central questions in seagrass ecology is the understanding of how seagrass ecosystems will respond to climate change, in particular to the increased incidence of severe climatic events such as summer heatwaves^44,45^. Predictions have initially challenged their long-term survival^46–48^, although some recent studies are glimpsing a more optimistic future for these marine plants. These studies have demonstrated that seagrasses are phenotypically flexible organisms that display intraspecific variability in morphology and physiology in response to environmental changes (e.g.^49–51^). Furthermore, current findings on gene expression patterns in seagrasses revealed a wide phenotypic plasticity, which can play a key role in determining how species and phenotypes withstand climate change (e.g.^52–54^). So far, molecular studies on seagrasses remain limited and the complete genome has been sequenced only for two species of the *Zostera* genus^41,55^.

The epigenetic research on marine plants is still at the infancy to date. Just two studies have characterized methylation levels at specific gene loci of the species *Posidonia oceanica* in response to specific environmental conditions^56,57^. The level of global DNA methylation has also been seen to vary with tissue age, likely resulting from the interplay of developmental and light cues, and under heat stress and chronic low light^58–60^. Just very recently, methylome variation among ramets of the same genet have been reported in the seagrass *Zostera marina*, together with co-variation between DNA methylation and photosynthetic performance under experimental conditions, which suggests a link between epigenetic variations and phenotypic plasticity of the species^61^.

The general objective of the present study is to evaluate potential relationships between DNA methylation and environmentally-driven gene expression and phenotypic plasticity in marine plants. By exploring two species with different ecological and biological attributes, we expanded our analysis to contrasting life history strategies of seagrasses. In both species, we addressed historic gbM patterns and present-day whole genome methylation levels. The transcriptomes of the two species were examined through in silico analyses and the ratio of observed to expected CpG dinucleotides (CpG_O/E_) was used to predict methylation status of specific genes, on the basis of CpG evolutionary changes induced by the hypermutability of mC. In particular, we aimed: (i) to explore differences in the level of predicted gene body DNA methylation between housekeeping and environmental/stress responsive genes; (ii) to examine the potential link between gbM and transcriptional regulation, by comparing the transcriptome profiles of different species and ecotypes in relation to their thermal origin and to their response to heat stress. We hypothesized that environmentally flexible gene-expression patterns are associated with signatures of weak gbM, and that ecotypes with different thermo-tolerance would show distinct relationships between DNA methylation and gene-expression profiles. Finally, to complement the in silico analysis, (iii) we empirically characterize intra- and inter-specific differences in DNA methylation via colorimetric quantification of global DNA methylation.

## Results

### Evaluation of predicted gbM in seagrasses

**Figure 1:**
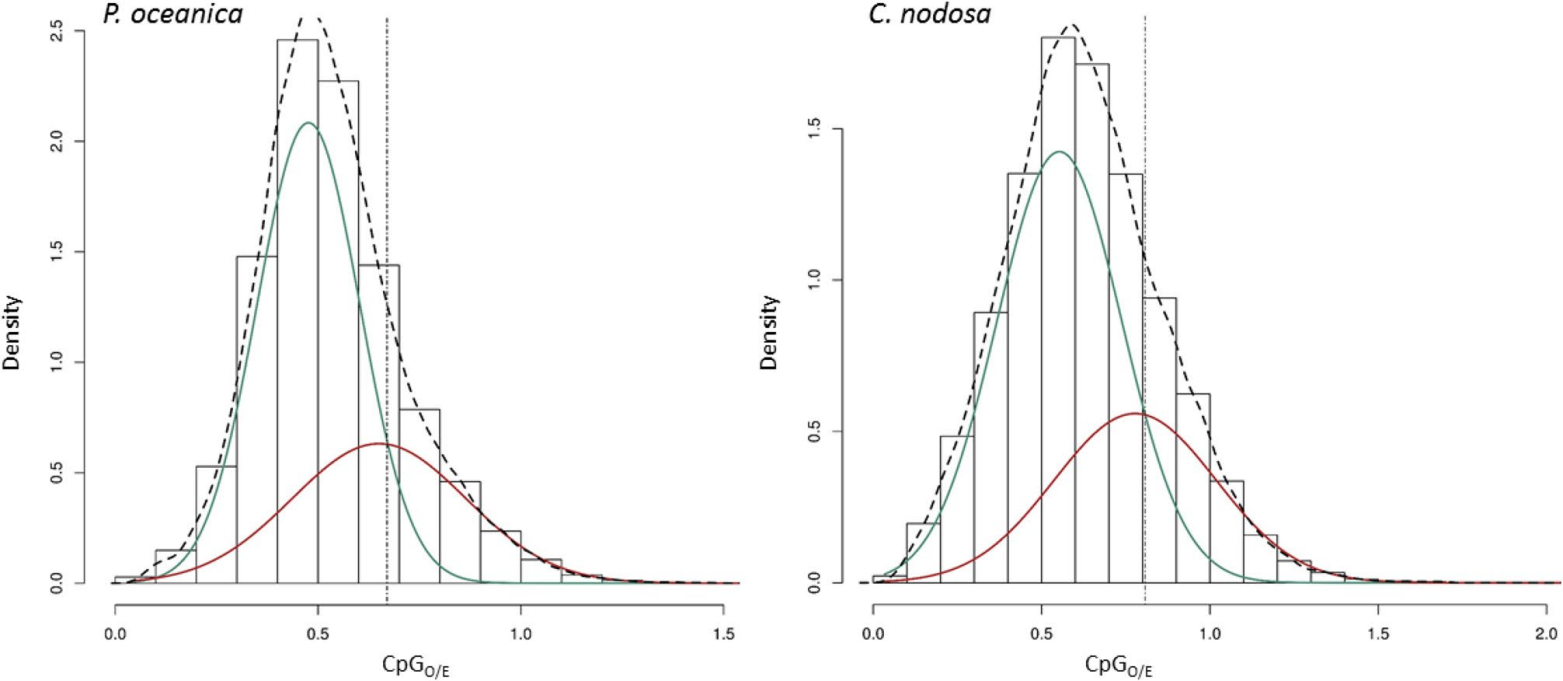
Density plots of CpG_O/E_ values from gene bodies of the seagrasses *P. oceanica* (left panel) and *C. nodosa* (right panel). Black dashed lines represent the unimodal CpG_O/E_ distribution, whereas the green and red solid lines represent low-CpG_O/E_ and high-CpG_O/E_ components respectively, as derived from the bimodal distribution provided by the 2-component Gaussian model. Dotted vertical lines indicate the intersection of the two fitted component curves.

Our in silico transcriptome analysis showed that predicted patterns of gene body DNA methylation, as measured by CpG_O/E_, were similar for the two seagrass species (Fig. 1). Bimodal mixture models showed a significant improvement in fit compared to the normal null model indicating that the distribution of CpG_O/E_ values for both species is better described by a mixture of two distinct Gaussian distributions (Table 1), although the visual examination of density plots suggests that bimodality is not very strong in either of the species (Fig. 1). The intersection point of the 2-component density curves was used to objectively attribute genes into two components: the ‘low-CpG component’ (biased toward hypermethylation; green line) and the ‘high-CpG component’ (biased toward hypomethylation; red line). The intersection of the two fitted component density curves was at 0.67 for *P. oceanica* and 0.80 for *C. nodosa*. A large number of *P. oceanica* (80.06%) and *C. nodosa* (78.94%) annotated genes were depleted in CpG dinucleotides, suggesting a considerably higher number of predicted hypermethylated genes (CpG_O/E_ depletion).

**Table 1:** Distribution parameters of the mixture models of CpG_O/E_ in the two seagrass transcriptomes. Higher log-likelihood statistics indicates the model that better fits the density distribution.

Species	Lambda		Mu		Sigma		Log-likelihood (k = 1)	Log-likelihood (k = 2)
	Comp-1	Comp-2	Comp-1	Comp-2	Comp-1	Comp-2		
*P. oceanica*	0.661	0.339	0.476	0.650	0.127	0.214	18,741	21,138
*C. nodosa*	0.663	0.337	0.553	0.778	0.186	0.241	1712	2156

### Relationship between gbM and gene function

**Figure 2:**
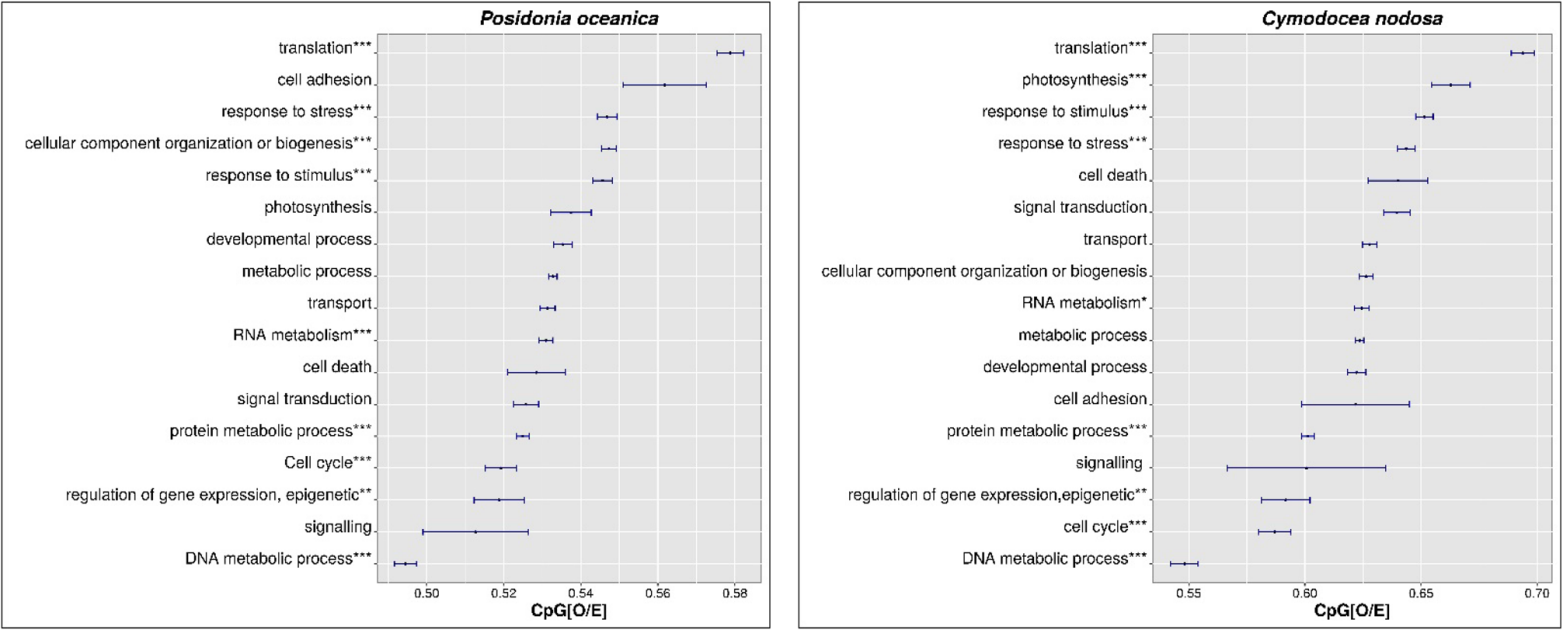
Variation of CpG_O/E_ among genes assigned to different GO Slim terms (biological processes) in *P. oceanica* (left panel) and *C. nodosa* (right panel). Points represent mean CpG_O/E_ for the corresponding biological processes (± standard error). Asterisks indicate significance of enrichment in the low- or high-CpG components (* < 0.05, ** < 0.01, *** < 0.001; Fisher’s exact test).

Both species showed similar patterns of gbM in relation to broad classes of biological processes (Fig. 2). Two distributions were comprised of functionally distinct gene classes in *P. oceanica* and *C. nodosa* and several biological processes showed CpG_O/E_ ratios that were significantly different (Fisher's exact test; FDR < 0.05), indicating that genes with lower-CpG_O/E_ and higher-CpG_O/E_ were involved in different biological functions. In both species, the top five biological processes with lower CpG_O/E_ were protein metabolic process, cell cycle, DNA metabolic process (all p < 0.0001), regulation of gene expression (p < 0.001) and signaling (ns). High-ranked biological processes with the highest mean CpG_O/E_ were a bit more variable between both species. However, genes with higher CpG_O/E_ ratios showed significant enrichment of terms associated to translation, stress response and response to stimulus in both seagrasses (p < 0.0001). Photosynthesis was also included within the high-ranked biological processes, ranking second and sixth in the list of *P. oceanica* and *C. nodosa*, respectively (Fig. 2).

### Relationships between gbM and gene expression patterns

#### Effects of plants’ thermal origin

Differences in gene expression that were distinctive of the thermal origin (warm *vs* cold) of *P. oceanica* and *C. nodosa* plants were explored by grouping the samples of each species according to their origin, regardless of the experimental treatment. The number of differentially expressed genes (DEGs) in the comparison between warm and cold populations was much higher in *P. oceanica* (4,465) than in *C. nodosa* (1,009), representing 3.31% and 1.23% of the total number of genes included in the species transcriptomes, respectively (Table 2).

**Table 2:** Number of differentially expressed genes (FDR < 0.05) in response to thermal origin of plants (warm-plants *vs* cold-plants), experimental treatment (i.e. warming; controls *vs* heated) and population-specific response to experimental warming (controls *vs* heated of cold- and warm-plants, separately). In parenthesis is shown the percentage that the number DEGs represent against the total number of genes of each species’ transcriptome.

	*P. oceanica*	*C. nodosa*
**Annotated genes with expression**	20,083 (14.89%)	12,230 (14.90%)
**Thermal origin**	4,465 (3.31%)	1,009 (1.23%)
**Experimental treatment**		
All samples	390 (0.29%)	1,870 (2.28%)
Cold-plants only	860 (0.64%)	1,614 (1.97%)
Warm-plants only	413 (2.06%)	1,071 (8.76%)

**Figure 3:**
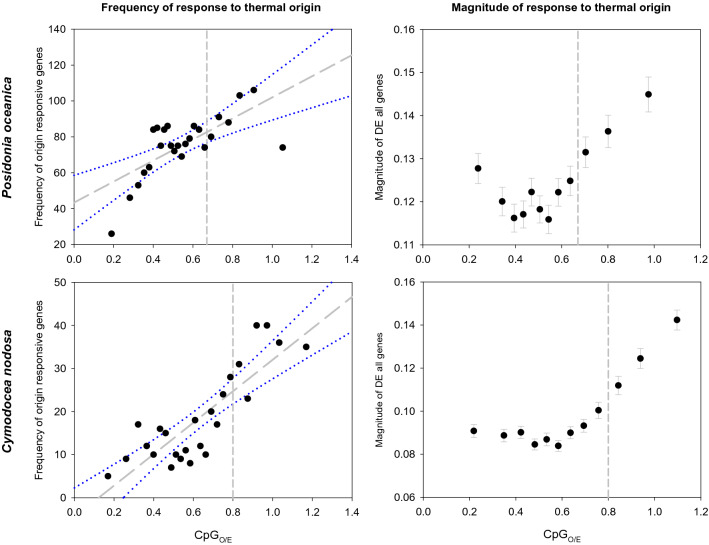
Relationship between CpG_O/E_ and gene responsiveness associated to the thermal origin of *P. oceanica* (upper panels) and *C. nodosa* (lower panels) plants. Frequency of plant origin flexible genes (left panels): each data point represents the number of DEGs (FDR < 0.05) responding to the thermal origin of plants within each of the 25 CpG_O/E_ bins and the mean CpG_O/E_ for the bin. Magnitude of response to thermal origin of plants (right panels): magnitude of differential expression for each annotated gene. Magnitude points represent the log mean differential expression value and standard error of the genes divided in 12 bins. Dashed vertical lines represent the intersection of the two fitted component curves of the bimodal distribution of CpG_O/E_ in each species. Solid grey lines and dotted blue lines represent the Pearson correlation line and the 95% confident intervals respectively.

Both species showed a progressive increase in the frequency of DEGs with the increase in CpG_O/E_ values (Fig. 3, left panels) as supported by their significant linear correlations (Pearson; N = 25, p < 0.001, R = 0.699 and 0.844 for *P. oceanica* and *C. nodosa*). Indeed, the number of DEGs in lower-CpG_O/E_ bins was significantly lower than in higher-CpG_O/E_ bins (t-test; p < 0.05 for *P. oceanica* and p < 0.001 for *C. nodosa*). In *P. oceanica,* the average number of DE genes was 71 (± 15; SD) for “low-CpG”, and 90 (± 12; SD) for “high-CpG” genes; while the corresponding values for *C. nodosa* were 14 (± 6; SD) and 34 (± 6; SD) respectively.

The magnitude of the expression difference between samples from different thermal origin also generally increased with the increase of CpG_O/E_ in both species (Fig. 3, right panels), although this relationship was observed mainly for CpG_O/E_ values > 0.5. Mean expression levels varied significantly across CpG_O/E_ (ANOVA; p < 0.001) and were positively correlated with CpG_O/E_ (Spearman rank; N = 12, p < 0.01, R = 0.755 and 0.843 for *P. oceanica* and *C. nodosa*). The differential expression due to plant origin increased sharply within the high-CpG_O/E_ component. This component showed a log mean magnitude of 0.138 and 0.126 in *P. oceanica* and *C. nodosa* respectively, while the corresponding magnitudes of genes from the low-CpG_O/E_ component were of 0.120 and 0.090. Mean magnitudes of expression of genes from the bins of each component were significantly different in both species (t-test; p < 0.001).

#### Species specific effects of experimental warming

**Figure 4:**
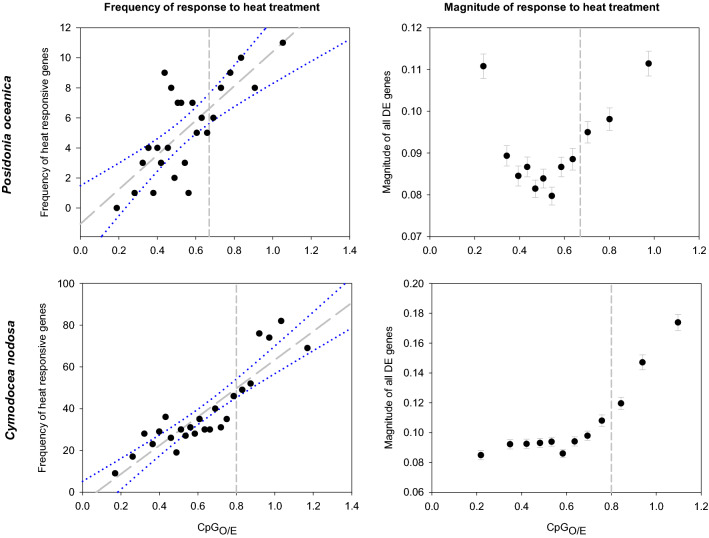
Relationship between CpG_O/E_ and gene responsiveness associated to the heat treatment in *P. oceanica* (upper panels) and *C. nodosa* (lower panels). Frequency of heat flexible genes (left panels): each data point represents the number of DEGs (FDR < 0.05) responding to the heat treatment within each of the 25 CpG_O/E_ bins. Magnitude of response to heat exposure (right panels): magnitude of differential expression for each annotated gene. Magnitude points represent the log mean differential expression value and standard error of the genes divided in 12 bins. Dashed vertical lines represent the intersection of the two fitted component curves of the bimodal distribution of CpG_O/E_ in each species. Solid grey lines and dotted blue lines represent the Pearson correlation line and the 95% confident intervals respectively.

When sample of each species were grouped according to the experimental treatment regardless the thermal origin of plants, 390 and 1,870 DEGs (i.e. 0.29% and 2.28% of the total number of genes in the corresponding transcriptomes) were found between control and heated plants of *P. oceanica* and *C. nodosa*, respectively (Table 2). Heat-responsive genes were overrepresented within the high-CpG_O/E_ component in both species, with average values that were the double that of values observed in the low-CpG_O/E_ component (Fig. 4, left panels). In particular, the average frequency of *P. oceanica* heat-responsive genes per bin was 4 (± 2; SD) for the low-CpG_O/E_ component and 9 (± 2; SD) for the high-CpG_O/E_ component. In *C. nodosa*, the number of genes significantly responding to the heat treatment was much higher with respect to *P. oceanica*, and showed an average of 29 per bin (± 8; SD) for the low-CpG_O/E_ component and 67 per bin (± 13; SD) for the high-CpG_O/E_ component. Both species showed a significant positive correlation between CpG_O/E_ values and frequency of DEGs per bin (N = 25, p < 0.001, R = 0.752 and 0.892 for *P. oceanica* and *C. nodosa*) and mean DEGs frequency was significant different between bins from the low- and high-CpG components (t-test; p < 0.001).

The magnitude of differential expression in response to heat (i.e. experimental treatment) varied significantly across CpG_O/E_ in both seagrasses (ANOVA; p < 0.001), being significantly higher in genes within the high-CpG_O/E_ component than in genes from the low-CpG_O/E_ component (t-test; p < 0.05 for *P. oceanica* and p < 0.001 for *C. nodosa*) (Fig. 4, right panels). In both species, the magnitude of expression of warming-responsive genes increased steeply within the high-CpG_O/E_ component. However, the correlation between CpG_O/E_ and magnitude of expression was only significant in *C. nodosa* (N = 12, R = 0.872, p < 0.001), since in *P. oceanica* (N = 12, R = 0.316, p = 0.317) the average expression of genes from the first bin (i.e. lowest CpG_O/E_) was similar to the magnitude of genes from the highest CpG_O/E_ bin.

#### Population-specific response to warming treatment

**Figure 5:**
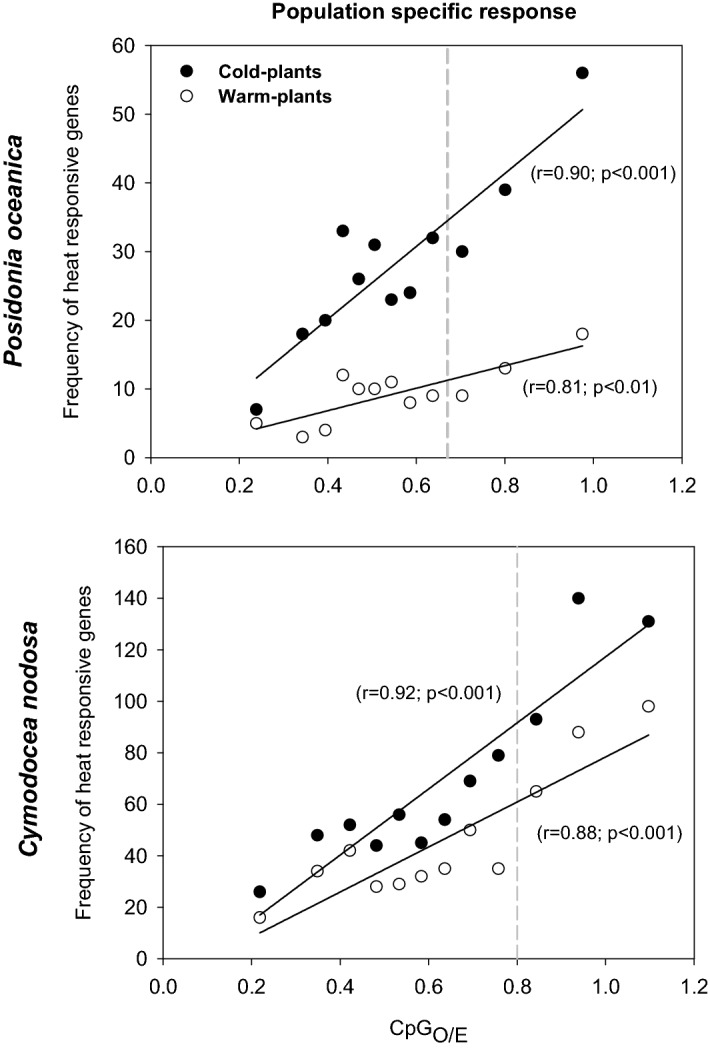
Relationship between CpG_O/E_ and frequency of heat responsive genes of *P. oceanica* (upper panels) and *C. nodosa* (lower panels) cold- (solid circles) and warm-plants (empty circles). Frequency of heat flexible genes: each data point represents the number of DEGs (FDR < 0.05) responding to the thermal treatment within each of CpG_O/E_ bins and the mean CpG_O/E_ for the bin.

At a smaller scale, we also examined the CpG_O/E_ composition of gene bodies in relation to differential gene expression under heat stress in plants of both species from the two different thermal origins (cold-plants *vs* warm-plants). In the four cases, the frequency of DEGs progressively increased with the increase of CpG_O/E_ levels (Fig. 5), and both parameters showed a significant linear tendency. In *P. oceanica*, DEGs frequency of cold-plants was significantly higher than in warm-plants (t-test, p < 0.001), but similar in the case of *C. nodosa* (t-test, p = 0.070). The slope of the linear adjustment of *P. oceanica* cold-plants, in fact, was 3.2 fold higher than the slope of warm-plants, while the difference was only of 1.5 for *C. nodosa* cold- and warm-plants.

The functional analysis of DEGs from the high-CpG_O/E_ component of *P. oceanica* showed a higher number of enriched GO terms (i.e. biological processes; FDR < 0.001) in cold-plants with respect to warm-plants (176 *vs* 38; Supplementary Table S1a, b). The ten most significant Biological Processes (i.e. lower FDR values) were common in plants from both populations; however, only cold-plants were enriched in the GO terms “response to external stimulus” (GO: 0009605), “response to abiotic stimulus” and (GO:0009628) and “response to stress” (GO:0006950), which match with the higher level of physiological stress experienced by these plants during the warming exposure^62^. These plants were also exclusively enriched in the biological processes “generation of precursor metabolites and energy” (GO:1901135) and “carbohydrate derivative metabolic process” (GO:0006091), reflecting the impaired energetic status (i.e. 20% reduced starch content) only reported in this experiment for *P. oceanica* cold-plants^62^. In addition, “cell wall organization or biogenesis” (GO:0071554) and “cellular lipid metabolic processes” (GO:0044255) were other exclusive enriched processes in cold-plants, which coincides with their incomplete lipid readjustment for cell wall and membrane fluidity acclimation to warming^63^. For its part, the functional analysis of DEGs from the high-CpG_O/E_ component of *C. nodosa* showed more uniform results between cold- and warm-plants in terms of number of GO-terms (170 and 128, respectively), in accordance with the similar DEGs frequency commented above. The enriched biological processes were quite similar between plants from both populations, with 84 shared GO-terms (Table S1c, d), likely reflecting the higher heat tolerance of the species and the similar heat responses of both *C. nodosa* ecotypes^62^. The main difference between both *C. nodosa* ecotypes was the inability of cold-plants to compensate heat-enhanced respiratory activity through a proportional increase in photosynthetic rates to avoid plant carbon imbalances^62^. Accordingly, “photosynthesis” (GO:0015979) and “photosynthesis, light reaction” (GO:0019684) were much more significantly enriched in cold-plants (5.46E−23 and 5.11E−18, respectively) than in warm-plants (8.91E−08 and 2.76E−04, respectively).

### Global DNA methylation

**Figure 6:**
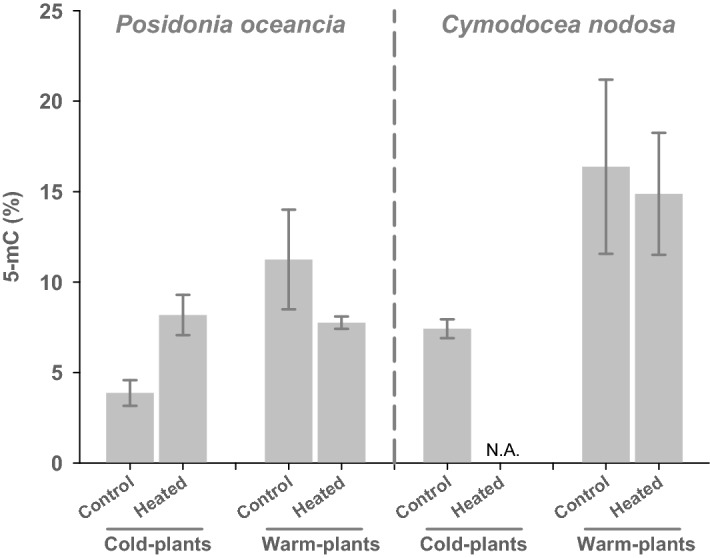
Global genomic methylation levels in *P. oceanica* and *C. nodosa* cold- and warm-adapted ecotypes from the control and heated treatments at the end of the warming exposure. Methylation values were calculated as percentage of the methylated-C in total genomic DNA.

The analysis of global DNA methylation (5-mC) showed constitutive differences between plants of both species from different origins (Fig. 6). The percentage of methylated-C in total genomic DNA of control warm-plants was significantly higher than that of control cold-plants (p < 0.05). In response to heat stress, contrasting responses were observed between *P. oceanica* cold- and warm-plants. While cold-plants doubled the percentage of 5-mC (p < 0.05), warm-plants did not significantly modify their global DNA methylation levels. Regarding *C. nodosa* warm-plants, they did not modify their percentage of 5-mC in response to heat stress exposure, and unfortunately the response of cold-plants could not be characterized due to the lack of plant material available for analysis in this particular treatment.

## Discussion

This study provides one of the first insights into the potential relationship between DNA methylation patterns and gene expression plasticity in marine plants. The two species here studied, *P. oceanica* and *C. nodosa*, have shown high levels of gene body methylation (gbM), differences in the level of total DNA methylation (5-mC) according to the origin of plants (e.g. latitudinal gradient of distribution) and the ability to modify global methylation levels in response to environmental changes (i.e. heat stress). We also found significant relationships between gbM and gene functionality, as well as between gbM and transcriptome profile under different environmental conditions, suggesting a possible link between epigenetic mechanisms and seagrass stress responses, as previously described in other organisms (e.g.^64–67^).

Our in silico transcriptome analyses of CpG_O/E_ ratios have shown that genes of both *P. oceanica* and *C. nodosa* could be divided into two groups, according to their patterns of DNA methylation. Different CpG_O/E_ ratio distributions have been recently described in eukaryotes^38^, including a highly marked bimodality in diverse invertebrate taxa, through similar analyses^13,24,25,40^. Gene G + C content seems to be heterogeneous in plants and bimodal and unimodal distributions have been observed through different genome-wide epigenetic methods (e.g. bisulfite sequencing, methylation-sensitive markers)^68,69^. Based on the consideration that gbM is evolutionarily conserved, it has been proposed that gbM may cause the bimodality of G + C content among grass (and monocot) genes^20^.

Our results are in accordance with the high levels of genic DNA methylation described within plant genomes^14,17,21,70^. Nevertheless, the percentage of gbM genes varies widely within and among species due to unknown reasons^14,21,26^. Most of *P. oceanica* and *C. nodosa* transcripts were depleted in CpG dinucleotides (i.e. CpG_O/E_ < 1.0), suggesting that the bulk of their genes possess high levels of methylation.

Our analysis also indicated that in both seagrass species, genes with lower CpG_O/E_ (predicted to be hyper-methylated) were significantly enriched for GO terms related to general metabolic functions, while high CpG_O/E_ genes (predicted to be hypo-methylated) showed a significant enrichment of terms associated to dynamic biological functions. These results are consistent with distinctive functional enrichment patterns observed in other plant species^15,20,23,26,70^ and invertebrates^18,24,25,32^. Hyper-methylation of ubiquitously expressed genes has been proposed as a homeostatic mechanism for limiting transcriptional regulation opportunities in critical genes (i.e. housekeeping). These genes are typically constitutively expressed and function to maintain basic cellular functions (e.g.^17,28,30^). On the contrary, genes with significantly lower levels of methylation tend to be the ones with inducible expression and more likely to exhibit tissue and/or environmental condition specificity^24,30–32^. Unmethylated or sparsely methylated genes have been proposed to passively facilitate a greater variety of transcriptional opportunities including access to alternative transcription start sites, increasing sequence mutations, exon skipping and greater epigenetic flexibility through transient methylation^24,25,30,71^. Therefore, the fact that genes differentially expressed in response to experimental warming were highly related to the high-CpG_**O/**E_ component in *P. oceanica* and *C. nodosa* is in line with the hypothesis that weak gbM facilitates environmentally responsive expression. However, the functional consequence of gbM on gene expression is controversial and still needs to be demonstrated since several other factors, including gene length, the number of transcription factors regulating the genes and the chromatin environment are potentially involved in facilitating expression variability^72,21^.

Genes with differential expression depending on plants’ origin tended toward high CpG_O/E_, suggesting that putative hypo-methylated genes are more likely to be differentially expressed between plants from different native thermal environments. Similar results have been also shown in natural ecological contexts of the reef-building coral *Acropora millepora*, where genes with differential expression based on coral’s origin were enriched in the low methylation component^32^, reinforcing the possible link between weak gbM and gene expression (phenotypic) plasticity. As suggested in corals, one possible explanation for the origin-specific expression differences could be the genetic divergence among the studied populations^32^. A strong genetic structure along the latitudinal gradient of distribution of *P. oceanica* in the western Mediterranean has been reported^73^ and accordingly, our experimental *P. oceanica* populations were genetically distinct^62^. In *C. nodosa*, however, such genetic pattern is not as strong within the western Mediterranean^74^.

Populations of the two species from the different thermal regimes showed contrasting levels of global genomic cytosine methylation (5-mC), with higher levels in warm-adapted with respect to cold-adapted plants. DNA methylation variations can result from stochastic epimutations, environmental induction or genetic control, which at the same time can be further shaped by natural selection and genetic drift^22,75–77^. Therefore, the interpretation of these epigenetic patterns is not straightforward in the studied populations since at the same time they naturally experience contrasting environmental conditions (e.g. temperature) and are genetically differentiated. In natural populations of the model species *Arabidopsis thaliana,* DNA methylation has shown striking correlations with the place of origin and its climate. In particular, the latitudinal distribution of plants was strongly correlated with methylation levels in transposable elements^66^ and in gene bodies^65^. This might suggest that temperature compensation has evolved in the natural range of the species favoring local adaptation. In accordance with this hypothesis, thermal adaptation along the latitudinal gradient of the species distribution has been evidenced in *P. oceanica*^78^, and the existence of local adaptation was recently suggested for *C. nodosa*, though in particular between Atlantic (Canary Archipelago) and Mediterranean populations^79^. In our analysis, this is further supported by the high number of genes differentially expressed on the basis of plants’ origin in *P. oceanica* with respect to *C. nodosa* (4,465 *vs* 1,009). Moreover, in our *P. oceanica* plants, thermal tolerance was previously shown to be higher in ecotypes from warm waters (i.e. southern latitudes) in respect to ecotypes from cold waters, which was interpreted as the result of local adaptation^62,63^. In *C. nodosa*, instead, plants from contrasting thermal environments exhibited similar tolerance to experimental warming. These differences in thermal tolerance were also further supported by our analysis since the response to warming was markedly different between the two *P. oceanica* (warm- and cold-plants) but similar between the two *C. nodosa* ecotypes (Fig. 5).

After the warming exposure, warm-adapted ecotypes of both species did not significantly modify their global methylation levels, but interestingly methylation levels increased in cold-adapted *P. oceanica* ecotypes (no data from *C. nodosa* cold-plants). These results suggest that plants from different thermal origins show different epigenetic responses likely to cope better with increased temperatures. Several studies have revealed that environmental stress can result in an increase or decrease in cytosine methylation throughout the genome and at specific loci, to mediate environmentally-responsive and stress-responsive gene expression^56,80–83^. Nevertheless, the interaction can also be the other way round, since it is plausible that changes in cytosine methylation, as stated above, were actually the result of changes in gene expression under stressful conditions^22,84^. In accordance with our results, cytosine methylation increased more in heat-sensitive genotypes of *Brassica napus* under increased temperatures than in heat-tolerant genotypes, likely to respond and adapt to heat-stress^80^. In seagrasses, global DNA hyper-methylation and methylation changes in target functional genes of *P. oceanica* have been shown under increased cadmium levels and reduced light conditions^56,57,60^, and have been proposed to be at the basis of the species acclimation to heat stress^54,58^. *P. oceanica* cold-plants not only changed DNA methylation levels during the warming exposure, but they also showed the modulation of genes involved in epigenetic processes commonly used by plants for the transcriptional stimulation of stress tolerance and flowering as a possible mechanism to survive and optimize reproductive success under stress conditions^85^. A very recent paper showed that the methylome of the seagrass *Z. marina* is flexible and responds directly to environmental changes induced by plant cultured under laboratory conditions and to heat stress^61^. Authors suggest that the co-variation in DNA methylation and photosynthetic performance may be linked via gene expression because methylation patterns varied in functionally relevant genes involved in photosynthesis, and in the repair and prevention of heat-induced protein damage. In consequence, this suggests that in marine plants DNA is amenable to epigenetic modulation through methylation. Although we cannot discriminate whether this is the cause or the consequence of the activation of heat-stress responsive genes, we can assume that it could play a key role in seagrasses to cope with and adapt to seawater warming. In fact, the modulation of methylation-related genes in response to heat stress has recently being suggested to be at the basis of thermal stress memory in seagrasses with the potential to accelerate the adaptation of marine plants to the ongoing climate change^86^.

The increase of global DNA methylation (empirical determination) after warming exposure in cold-adapted *P. oceanica* ecotypes could be somehow in contrast with results obtained from in silico normalized CpG content (CpG_**O/**E_) analysis. Indeed, most of DEGs under heat stress belonged to the high-CpG component in both species, and thus they are predicted to exhibit low gbM. Nevertheless, CpG_**O/**E_ does not necessarily correspond to the actual level of whole genome methylation, since cytosine methylation occurs within CG, CHG and CHH motifs^87^. Moreover, the global methylation analysis presented here does not discriminate between intra and inter-genic cytosine methylation, and the increase in DNA methylation could have occurred in inter-genic heterochromatic regions (e.g. repetitive, transposable elements (TEs)), which actually represent the bulk of methylated genomic sites in plants^87^ and/or in untranscribed regulative regions (e.g. gene promoters). In particular, a higher level of cytosine methylation changes around TEs, in respect to other genomic regions, has been described in plants following abiotic stress events (e.g.^88^). Overall, our data may suggest that plant’s stress response could occur in connection with both hypomethylation of responsive genes (gene body methylation) and hypermethylation of regulative regions and/or TEs^84^ (non-genic methylation).

In conclusion, and in agreement with some previous observations (e.g.^24,29–32^) our results support the hypothesis that gene body methylation is associated with gene expression flexibility: low-gbM genes are much more likely to be differentially expressed in response to environmental stimuli than high-gbM genes. This is consistent with the idea that high gbM may function to suppress flexibility in transcriptional regulation. However, we acknowledge that our evidence is not based on direct estimations of DNA methylation at specific loci or throughout the genome, and that the role of gbM in regulating gene expression is still under an intensive debate. GbM could also be passively induced and other molecular processes could be responsible of its regulation^22,72^.Interestingly, we show that the same association with gbM exists for genes that are differentially expressed between populations from different origins. This suggests that low-gbM genes could not only be more inducible, but their expression may also evolve more rapidly, pointing to a more relevant role in population divergence and local adaptation. However, further experiments are certainly needed to infer about the role of gbM in phenotypic plasticity and adaptive capacity of marine plants. Experimental removal of DNA methylation using specific enzymes^89^ or experimental modification of methylation patterns through genome editing methods (e.g. via CRISPR-Cas system^90^), could be critically useful to identify eventual causal relationships between methylation changes under stress and adaptive phenotypic changes.

## Methods

### Target species and experimental plants

In this study, we compared an opportunistic and a persistent seagrass species that differ in plant size, life expectancy and reproductive characteristics. *P. oceanica* is one of the slowest-growing and longest-living plants on earth, with predominant clonal growth and whose extensive and permanent populations are considered one of the main climax stages of the Mediterranean coastal environment^91^. In contrast, *C. nodosa* is a fast-growing colonizer species of medium-size, characterized by high rates of both sexual reproduction and clonal propagation through rhizome elongation, and whose populations show a marked demographic seasonal pattern^92^. Based on these striking inter-specific differences, *C. nodosa* is considered to be more plastic to environmental changes, including light reduction^93^, salinity increase^51^ and temperature rise^50^.

The experimental plants used in this study for the analysis of genic CG methylation patterns, total DNA methylation and transcriptomic changes came from the experiment described in^62,63^. Briefly, early summer plants of each species were collected from two different populations that were ca. 700 km apart and that experienced different natural thermal environments (up to 6 °C of difference in summer sea-surface temperature). Apical plant fragments bearing at least 10 interconnected vertical shoots were individually transplanted in plastic pots and four of these pots were randomly distributed in each tank of the experimental mesocosm systems of the Spanish Institute of Oceanography. Twelve independent 500-L tanks were used for *P. oceanica* and twelve 100-L tanks for *C. nodosa*, with half of the tanks containing plants from the cold-environment population (cold-plants) and the other half plants from the warmer-environment population (warm-plants). After a 3-week acclimation period, and for each of the species and ecotypes, the temperature in three of the six tanks was progressively increased of 4 °C (rate of 0.5 °C day^−1^) above mean summer levels (control temperature) and maintained during 6 weeks to simulate a marine summer heatwave. Thus, the experiment followed a factorial design with population (cold *versus* warm) and experimental treatment (control *versus* high temperature) as factors and three independent replicates per group. After the warming period, a 7 cm leaf segment from the middle portion of the youngest and fully developed leaf was collected (14:00 h) from one randomly selected shoot per pot. Leaf segments were conserved in RNAlater for RNA-seq analysis or flash frozen in liquid nitrogen for total DNA methylation analysis. All tissues were stored at − 80 °C until RNA and DNA extraction. Leaf pieces of similar biomass (25 mg W.W.) from the four pots of each tank were pooled (*n* = 3 per treatment) before extractions.

### DNA extraction and global DNA methylation analysis

Genomic DNA was extracted from leaf tissues with NucleoSpin® Plant II kit (Macherey–Nagel) and the quality assessed through 1.0% (w/v) agarose 0.5X TBE gel (0.5 mg/mL EtBr). DNA purity was measured using a NanoDrop® ND-1000 Spectrophotometer (Thermo Fisher Scientific) and concentration determined by using the Qubit 2.0 Fluorometer (Thermo Fisher Scientific). Global DNA methylation was assessed colorimetrically in duplicate by an ELISA-like reaction with the MethylFlash™ Methylated DNA Quantification Kit (Epigentek Inc.), and reported as % of 5-mC methylated DNA relative to the input DNA quantity. Fifty nanograms of DNA per sample were analyzed. Absorbance at 450 nm was assayed using a Multiskan™ FC Microplate Photometer (Thermo Fisher Scientific). Significant differences in the level of global methylation among samples were explored through ANOVA analysis using the statistical package STATISTICA (StatSoft, Inc.v. 10).

### RNA extraction and sequencing

Leaf RNA was extracted using the Aurum Total RNA kit (Bio-Rad). The quality and quantity of the extractions were assessed by using Nanodrop (Thermo Fisher Scientific), 1% agarose gel electrophoresis and a 2100 BioAnalyzer (Agilent). Twelve libraries of each species, six from each thermal-population, half of which were from control tanks and half from heated tanks, were sequenced on an Ion Proton™ sequencer and a total of 106,210,104 and 89,086,102 single-end reads of 85 bp in average were generated for *P. oceanica*^85^ and *C. nodosa*, respectively.

### Seagrass transcriptomic resources

We used transcriptome assemblies as a basis for gene body methylation analysis, since no genomic references are currently available for *P. oceanica* and *C. nodosa* (see Table 3 and Supplementary information). For *P. oceanica*, we used a transcriptome assembly that was recently generated^85^, which is the most complete assembly currently available. A description of the *P. oceanica* transcriptome construction and functional annotation can be found in^85^. For building the *C. nodosa* transcriptome, we updated a previously published transcriptome assembly^94^ with the transcriptome reads obtained in this study (77,222,158 (86.68% of raw reads) HQ single-end cleaned reads, see Supplementary information). The same procedure as in^85^ was followed for the functional annotation and characterization of the *C. nodosa* transcript set (Supplementary information).

**Table 3:** Summary statistics of *Posidonia oceanica*^85^ and *Cymodocea nodosa *de novo transcriptomes used in the present study. GC = the proportion of guanine and cytosine nucleotides among total nucleotides; N50 = the length of the longest contig such that all contigs of at least that length compose at least 50% of the bases of the assembly; RMBT = percentage of reads that mapped back to transcripts in each final assembly*; BUSCO: Benchmarking Universal Single-Copy Orthologs*.

	*P. oceanica*	*C. nodosa*
**Number of transcripts**	225,579	117,740
**Number of genes**	134,863	82,095
**GC**	41.02	41.18
**N50**	1.993	1,819
**Mean (bp)**	990	933.55
**Median (bp)**	466	455
**Minimum contig length (bp)**	201	201
**Maximum contig length (bp)**	17,138	18,275
**Number of transcripts > 1,000 bp**	90,311 (40.04%)	45,845 (38.94%)
**RMBT**	74,95%	64.09%
**BUSCO**		
Complete orthologs	88,2%	79.24%
Single-copy	34.9%	52.36%
Duplicated	53.3%	26.88%
Fragmented orthologs	5.1%	8.61%
Missing orthologs	97 6.7%	12.15%
Total orthologs	1,440	1,440
**Number of annotated transcripts**	65,388 (48.11%)	39,802 (33.80%)

### Evaluation of predicted germline DNA methylation patterns in seagrasses

We characterized CpG density in gene bodies through the CpG observed/expected ratio (CpG_O/E_). The CpG_O/E_ for each gene was defined as:

$${CpG}_{O/E}=\frac{number\,of\,CpG}{number\,of\,Cx\,number\,of\,G}x\frac{{l}^{2}}{l-1}$$

where *l* is the number of nucleotides in the gene to account for the effect of gene length on gbM^31,72^. In silico analysis was limited to annotated sequences in order to be confident that sequences were reported in the 5′ to 3′ direction.

To evaluate if CpG_O/E_ values of both transcriptomes were best described as a single distribution or as a mixture of different distributions, we used the package Mclust^95^ in R v3.4.1^96^. Bayesian information criterion (BIC) was used to compare the likelihood of Gaussian mixture models with different numbers of components. Subsequently, the normalmixEM function in the mixtools R package^97^ *was used* to fit and estimate the mixture distribution parameters. Mixture models were evaluated against the null single-component model by comparison of log-likelihood statistics.

### Relationship between gbM and gene function

To evaluate the variation of CpG_O/E_ within and among functional biological process of *P. oceanica* and *C. nodosa* genes, we summarized this set of annotations to 19 high level Gene Ontology (GO) biological processes list using the Map2Slim option in OWLTools (https://github.com/owlcollab/owltools). The GO terms were selected to match a subset of those analyzed in previous works^25,31,32^ as well as to better demonstrate the spread of “housekeeping” *versus* dynamic biological processes.

In order to assess if low-CpG and high-CpG genes were functionally distinct, we performed a GO biological process term enrichment using the fisher.test function in R v3.4.1, by comparing low-CpG and high-CpG genes separately with a background reference composed by all genes.

### Relation between gbM and transcription

Reads generated from the experimental plants of this study were individually mapped to the corresponding transcriptome using the Bowtie v.1.2 aligner^98^ and expression levels of each gene were quantified using the Expectation–Maximization (EM) method of RSEM v1.3.0^99^. Before differential expression (DE) analysis, very lowly expressed genes were removed keeping those genes that have at least a cpm (read/count per million) of 1 or greater for at least three samples (the size of the smallest group of replicates); subsequently, data were normalized to scale the raw library sizes. Statistical inference of differential expression at the gene level was performed by edgeR v3.16.5^100^ under a generalized linear model (GLM) approach.

For each species, DE analysis was firstly performed between ecotypes (i.e. cold-plants *vs* warm-plants, irrespective of their thermal treatment) to test for differences in gene expression that were distinctive of the populations of origin. DE comparison was also performed between control and heated plants of each species (irrespective of their thermal origin) to test the impact of seawater warming on gene expression. Finally, DE analysis was conducted for each of the ecotypes independently (control *vs* heated plants) to explore the potential role of gbM on the transcriptomic response (i.e. plasticity) of cold- and warm-plants to heat stress. Genes were considered differentially expressed at a significance of a false discovery rate (FDR) threshold corrected p value < 0.05. The magnitude of the expression difference for each DEG was calculated as log (mean expression Warm-plants/mean expression Cold-plants) for the plants origin comparison and, log (mean expression Heated plants/mean expression control plants) for the warming treatment comparison.

Trends between differential expression and CpG composition of genes were explored by plotting the frequency (i.e. number of DEGs) and mean differential expression (i.e. magnitude) values against predicted methylation. For each gene, expression was averaged across all samples. These data were then joined with CpG_O/E_ data and genes were divided into 25 (803 and 489 genes per quantile for *P. oceanica* and *C. nodosa*, respectively) and 12 (1.672 and 1.019 genes per quantile for *P. oceanica* and *C. nodosa*, respectively) equally sized quantiles based on CpG_O/E_, for the frequency and magnitude analysis, respectively. Mean expression for all genes in each quantile was then plotted against the mean CpG_O/E_ value for the quantile.

Relationships between CpG_O/E_ values and the frequency and magnitude of expression of DEGs were explored through Pearson product-moment correlation. Analysis of variance (ANOVA) was conducted to test if the magnitude of expression significantly differed among CpG_O/E_ bins. Significant differences in the number of DEGs or the magnitude of expression between the low-CpG component and the high-CpG component were assessed though *t*-test. A p-value cutoff of 0.05 was used in the statistical analyses that were performed using the statistical package STATISTICA (StatSoft, Inc.v. 10).

## Supplementary Information

Supplementary Information 1.

Supplementary Information 2.

## Data Availability

This study made use of publicly available transcriptome data. *P. oceanica* data are available at the Sequence Read Archive (SRA) at the National Center for Biotechnology Information (NCBI) under the Accession Number SRP126951 (RNA-Seq reads) and at DDBJ/EMBL/GenBank under the accession GGFN00000000. *C. nodosa* data are available at the ENA (European Nucleotide Archive) repository (http://www.ebi.ac.uk/ena) under Accession nos. HADH01000001-HADH01059478. RNA-Seq reads generated for this study are accessible at the Sequence Read Archive (SRA) at the National Center for Biotechnology Information (NCBI) under BioProject ID PRJNA548629.
