# Supplementary material for: Gene body DNA methylation in seagrasses: inter- and intraspecific differences and interaction with transcriptome plasticity under heat stress
Source: Sci Rep. 2021 Jul 12;11:14343. doi: 10.1038/s41598-021-93606-w (PMC8275578; doi:10.1038/s41598-021-93606-w)
Supplement: Supplementary file 1 — Supplementary Information 1. [file 41598_2021_93606_MOESM1_ESM.docx]

**Supporting Information for:**

**Gene body DNA methylation in seagrasses: inter- and intraspecific differences and interaction with transcriptome plasticity under heat stress**

Laura Entrambasaguas, Miriam Ruocco, Koen J.F. Verhoeven, Gabriele Procaccini, Lazaro Marín-Guirao

**Supporting methods**

**Transcriptomic resources of *Cymodocea nodosa***

Quality of sequence raw reads of *C. nodosa* plants from this study were checked with FastQC v0.11.5 (http://www.bioinformatics.babraham.ac.uk/projects/fastqc/). Reads with average quality scores lower than 20 and a minimum length less of 25 were discarded by using the fastx-toolkit v0.0.14 (http://hannonlab.cshl.edu/fastx_toolkit). Trimmed reads were checked for possible ribosomal contamination through BLASTn (E-value ≤ 1E−20) against 8 default ribosomal RNA (rRNA) databases using SortMeRNA v2.1 ^1^. A final FastQC run was performed on the remaining data to ensure the validity of preprocessing steps. High quality cleaned reads were assembled into a transcriptome using Trinity v.2.3.2 ^2^ with *in silico* read normalization, Kmer length 25 and parameters: “--max_memory 240G --seqTypefq --CPU 24 --min_kmer_cov 2”.

For constructing the *C. nodosa* transcriptome, we combined a previously published transcriptome ^3^ and the newly generated by the cleaned reads from the experimental plants from this study. Firstly, identical contigs from each assembly were removed using CD-HIT-EST v4.6.7 ^4^ with 100% identity and a word size of 10. Non-redundant sequences that were greater than 200 bp were kept. Both transcriptomes were subsequently combined and further processed by CD-HIT-EST with settings '-c 0.95 -n 10 -d 0 -M 220000 -T 32 -p 1 -g 1' in order to remove intra assembly redundancy.

The final *C. nodosa* meta-assembly statistics were generated using the 'TrinityStats.pl' script and calculating the number of contigs over 1,000 base pairs (bps) long. We further evaluated the quality of the assembly and the transcript contiguity in terms of read representation by mapping cleaned reads back to the set of transcript sequences with Bowtie v1.2 ^5^. We also assessed the completeness of the *P. oceanica* and *C. nodosa* transcriptomes using the Benchmarking Universal Single-Copy Orthologs 3.1.0 (BUSCO; ^6^) method with the Embryophyta database on both assemblies to determine the representation of conserved plant orthologs.

For the functional annotation of *C. nodosa* transcriptome, we searched homolog sequences using BLASTX v2.6.0+ ^7^ with E-value ≤ 1E−6 against UniProtKB/Swiss-Prot and NCBI non-redundant sequence (Nr) protein databases. Subsequently, transcripts with a positive BLAST hit were used to retrieve functional annotation from the GO (Gene Ontology; ^8^) database using Blast2GO ^9^. Whenever a gene contained multiple isoforms, the longest isoform was defined as the gene functional annotation and used in subsequent analysis (e.g. enrichment analysis).

**Transcriptomic resources of *Posidonia oceanica***

*P. oceanica* transcriptome used in this study was the one generated by ^10^. In brief, this transcriptome was the result of combining four individual assemblies from ^10-13^ into one merged assembly using Trinity v.2.3.2 ^2^ with *in-silico* read normalization, Kmer length 25 and parameters: “--max_memory 240G --normalize_reads --seqTypefq --CPU 24 --min_kmer_cov 2”. Subsequently, highly similar contigs were clustered by similarity using CD-HIT-EST with settings ‘-c 0.95 -n 10 -g 1 -M 220000 -T 24’.

In addition to the quality assessment of the final *P. oceanica* transcriptome performed by ^10^, we also assessed it completeness using the same procedure previously described for *C. nodosa*.

Finally, sequences were subjected to sequence similarity search against NCBI non-redundant sequence (Nr) and UniProtKB/Swiss-Prot databases using BLASTX v2.6.0+ with an E-value cutoff value of 10−6 and further annotated in Blast2Go.

**References**

1 Kopylova, E., Noé, L. & Touzet, H. SortMeRNA: fast and accurate filtering of ribosomal RNAs in metatranscriptomic data. *Bioinformatics* **28**, 3211-3217, doi:10.1093/bioinformatics/bts611 (2012).

2 Haas, B. J. *et al.* De novo transcript sequence reconstruction from RNA-seq using the Trinity platform for reference generation and analysis. *Nature protocols* **8**, 1494-1512, doi:10.1038/nprot.2013.084 (2013).

3 Ruocco, M. *et al.* Genomewide transcriptional reprogramming in the seagrass *Cymodocea nodosa* under experimental ocean acidification. *Molecular ecology* **26**, 4241-4259, doi:10.1111/mec.14204 (2017).

4 Huang, Y., Niu, B., Gao, Y., Fu, L. & Li, W. CD-HIT Suite: a web server for clustering and comparing biological sequences. *Bioinformatics* **26**, 680-682 (2010).

5 Langmead, B., Trapnell, C., Pop, M. & Salzberg, S. Ultrafast and memory-efficient alignment of short DNA sequences to the human genome. *Genome biology* **10**, R25 (2009).

6 Simão, F. A., Waterhouse, R. M., Ioannidis, P., Kriventseva, E. V. & Zdobnov, E. M. BUSCO: assessing genome assembly and annotation completeness with single-copy orthologs. *Bioinformatics* **31**, 3210-3212 (2015).

7 Altschul, S. F., Gertz, E. M., Agarwala, R., Schäffer, A. A. & Yu, Y.-K. PSI-BLAST pseudocounts and the minimum description length principle. *Nucleic acids research* **37**, 815-824 (2009).

8 Ashburner, M. *et al.* Gene ontology: tool for the unification of biology. The Gene Ontology Consortium. *Nature genetics* **25**, 25 - 29 (2000).

9 Conesa, A. *et al.* Blast2GO: a universal tool for annotation, visualization and analysis in functional genomics research. *Bioinformatics* **21**, 3674 - 3676 (2005).

10 Marín-Guirao, L., Entrambasaguas, L., Ruiz, J. M. & Procaccini, G. Heat-stress induced flowering can be a potential adaptive response to ocean warming for the iconic seagrass *Posidonia oceanica*. *Molecular ecology* **28**, 2486-2501, doi:10.1111/mec.15089 (2019).

11 D’Esposito, D. *et al.* Transcriptome characterisation and simple sequence repeat marker discovery in the seagrass Posidonia oceanica. *Scientific data* **3**, 160115 (2016).

12 Entrambasaguas, L. *et al.* Tissue-specific transcriptomic profiling provides new insights into the reproductive ecology and biology of the iconic seagrass species *Posidonia oceanica*. *Marine genomics* **35**, 51-61, doi:<https://doi.org/10.1016/j.margen.2017.05.006> (2017).

13 Marín-Guirao, L., Entrambasaguas, L., Dattolo, E., Ruiz, J. M. & Procaccini, G. Molecular Mechanisms behind the Physiological Resistance to Intense Transient Warming in an Iconic Marine Plant. *Frontiers in plant science* **8**, doi:10.3389/fpls.2017.01142 (2017).
